# Uniaxial orientation of P3HT film prepared by soft friction transfer method

**DOI:** 10.1038/s41598-017-05396-9

**Published:** 2017-07-11

**Authors:** Masayoshi Imanishi, Daisuke Kajiya, Tomoyuki Koganezawa, Ken-ichi Saitow

**Affiliations:** 1 Department of Chemistry, Graduate School of Science, Hiroshima University, 1-3-1 Kagamiyama, Higashi-hiroshima, Hiroshima, 739-8526 Japan; 2 Natural Science Center for Basic Research and Development (N-BARD), Hiroshima University, 1-3-1 Kagamiyama, Higashi-hiroshima, Hiroshima, 739-8526 Japan; 3 Japan Synchrotron Radiation Research Institute (JASRI), SPring-8, 1-1-1 Kouto, Sayo, Hyogo, 679-5198 Japan

## Abstract

The realization of room-temperature processes is an important factor in the development of flexible electronic devices composed of organic materials. In addition, a simple and cost-effective process is essential to produce stable working devices and to enhance the performance of a smart material for flexible, wearable, or stretchable-skin devices. Here, we present a soft friction transfer method for producing aligned polymer films; a glass substrate was mechanically brushed with a velvet fabric and poly(3-hexylthiophene) (P3HT) solution was then spin-coated on the substrate. A P3HT film with a uniaxial orientation was obtained in air at room temperature. The orientation factor was 17 times higher than that of a film prepared using a conventional friction transfer technique at a high temperature of 120 °C. In addition, an oriented film with a thickness of 40 nm was easily picked up and transferred to another substrate. The mechanism for orientation of the film was investigated using six experimental methods and theoretical calculation, and was thereby attributed to a chemical process, i.e., cellulose molecules attach to the substrate and act as a template for molecular alignment.

## Introduction

Optoelectronic properties can be improved by the anisotropy of molecules in a solid. The molecular orientation in conjugated polymers has attracted particular attention in the chemical research field and for practical applications^1–5^ Oriented polymer films have been utilized for flexible, lightweight, printed electronics, such as wearable or stretchable-skin devices^6, 7^, solar cells^8^, light-emitting diodes^9^, and transistors^10–12^. Several methods to prepare highly oriented polymer films have been reported, such as drawing, rubbing, friction transfer, alignment on a specific substrate, and directional crystallization^13–20^. Among these methods, friction transfer is a facile process that has been used to obtain polymer films with highly uniaxial orientation since it was first reported by Wittmann and Smith^20–26^. The friction transfer method is conducted by sliding a solid polymer material on a substrate heated at temperatures in the range of 140–180 °C. However, for flexible electronic devices to be integrated on a polymer substrate, such temperatures may be too high because the material typically has low heat resistance. Thus, a low-temperature process is crucial for the development of organic-based flexible electronic devices and would also be expected to be a cost-effective method from an industrial perspective.

**Figure 1:**
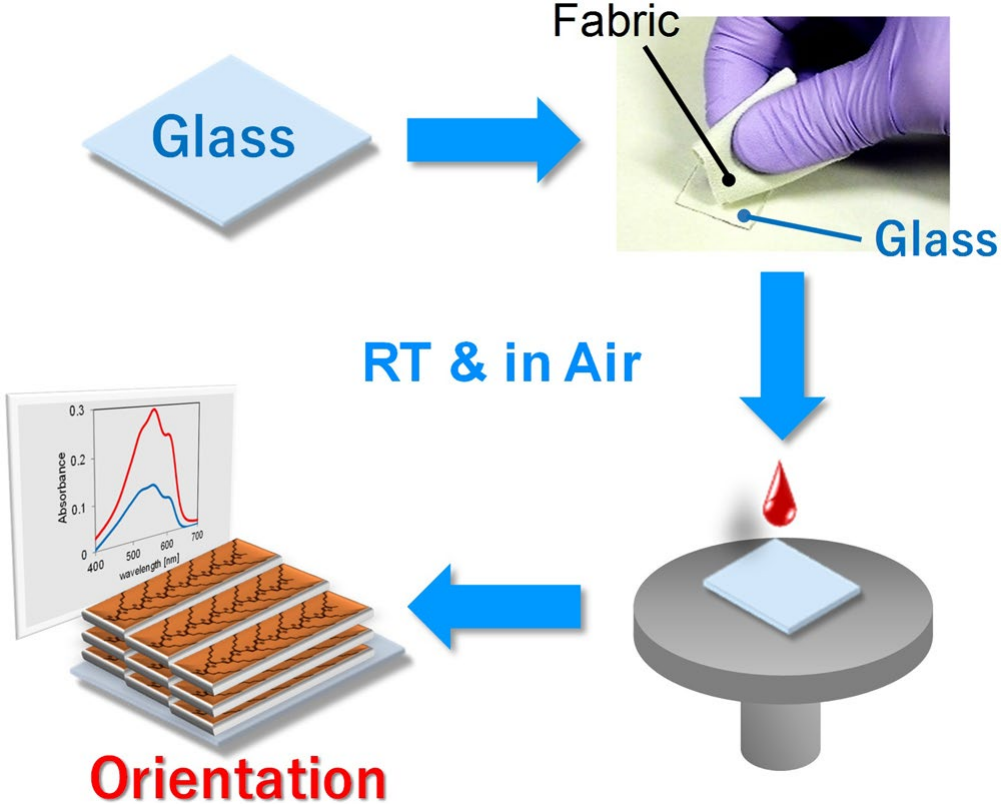
Schematic diagram of the soft friction transfer method conducted at room temperature (RT) and in air.

**Figure 2:**
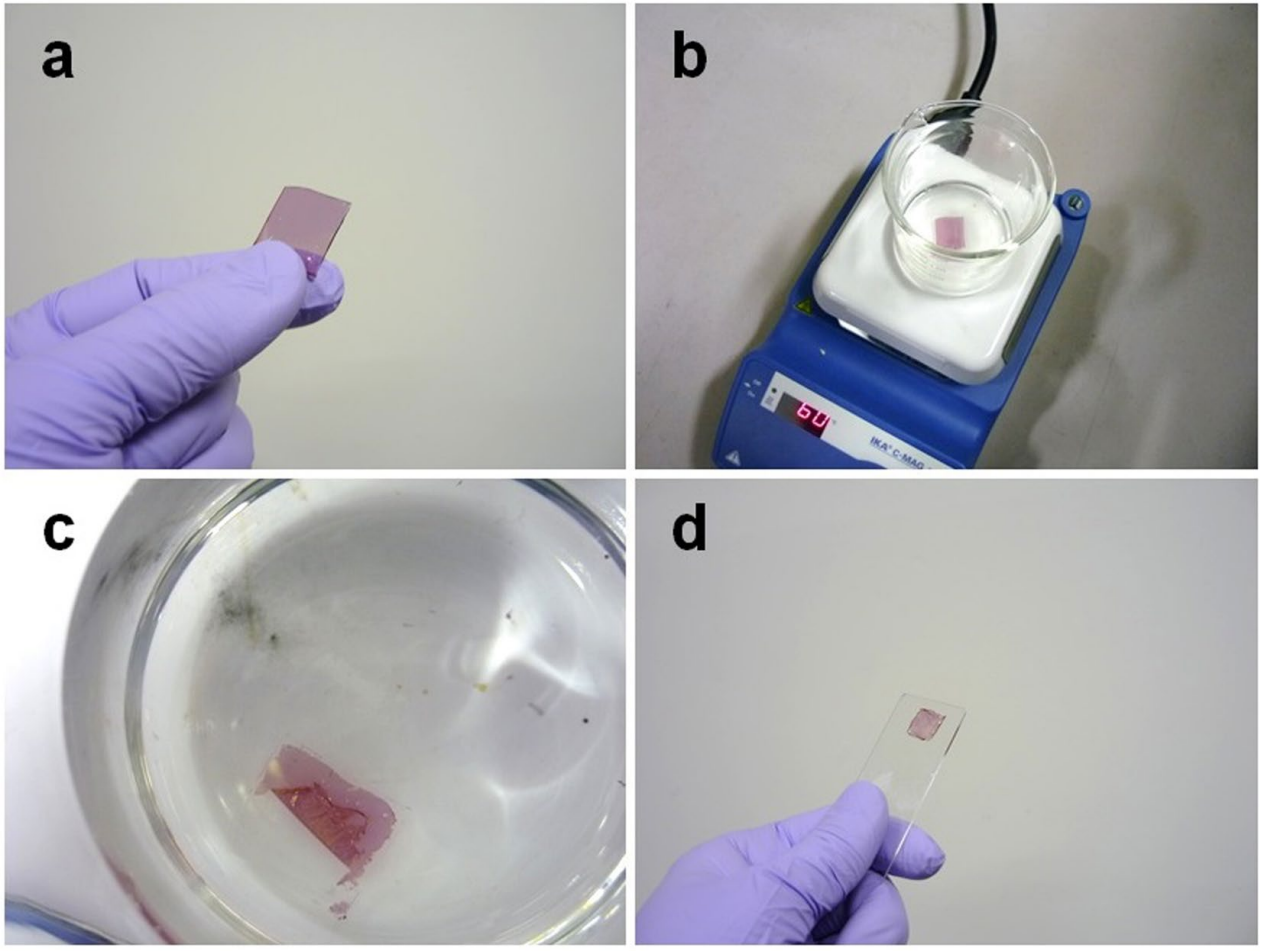
Photographs of uniaxial aligned 40 nm thick P3HT film picked up from a substrate. (**a**) P3HT film prepared using the soft friction transfer method on an ITO-coated glass substrate. (**b**) The film is immersed in water, (**c**) peeled from the substrate, and (**d**) then transferred to a glass slide as another substrate.

Facile methods have been previously reported, whereby molecules are oriented by drop casting and/or spin coating on a substrate mechanically brushed by a fabric^4, 27, 28^, a tissue paper^29^, or a paper^28^. Such methods can provide orientated films composed of small molecules^29^, liquid crystals^27, 28^, and oligomers^4^ at room temperature; however, the molecular orientation of polymers has not yet been reported. Here, we report the uniaxial alignment of a polymer film of poly(3-hexylthiophene) (P3HT) at room temperature, based on a similar approach. P3HT has been a popular polymer associated with flexible organic electronic devices such as tactile sensors^7^, field-effect transistors^30–33^, and solar cells^34–36^. The uniaxial aligned P3HT film is obtained by a two-step process: i) a substrate is mechanically brushed by a velvet fabric at room temperature, and ii) P3HT solution is then spin-coated on the substrate (Fig. 1). The resultant orientation factor was greater than that for a film prepared by conventional friction transfer on a Teflon plate heated at 120 °C. The soft friction transfer method presented here does not require high temperature, a glove box, or vacuum equipment, but provides uniaxial oriented films by a facile process that involves brushing a soft fabric on the substrate at room temperature. Cellulose is then transferred onto the substrate as a template for molecular alignment. The oriented P3HT film was picked up by immersing the glass substrate in water and transferring the film to another substrate (Fig. 2). Oriented films were also prepared using small molecules and polymers as well as different substrates. The orientation mechanism was analyzed using six experimental methods and by theoretical calculation. As a representative application, a polarized light-emitting device was demonstrated using an oriented polymer film prepared by soft friction transfer.

## Materials and Methods

An indium tin oxide (ITO)-coated glass substrate (FLAT-ITO, Geomatec) or glass substrate with a size of 2 × 2 cm^2^ was washed with a detergent solution (PK-LCG201, Parker Corp.) using a sonicator (3510 J, Branson), and then cleaned with an oxygen-plasma cleaner (Cute, Femto Science). The cleaned substrate was then brushed with a velvet fabric at a force-pressure of ca. 1–2 kgf/cm^2^. 30 μL of a 5 mg/mL solution of P3HT (Rieke Metals, P200) in chlorobenzene (Nacalai Tesque) was placed dropwise on the brushed ITO substrate and spin-coated at 1,000 rpm for 3 s and then at 2,000 rpm for 30 s. Finally, the P3HT film was dried in an air atmosphere and the film thickness was determined to be ca. 30 nm, using a confocal laser microscope (OLS4000, Shimadzu) equipped with a 100x objective. The P3HT thin film and ITO substrate itself were investigated using electronic absorption spectroscopy, grazing incidence X-ray diffraction (GIXD), scanning electron microscopy (SEM), atomic force microscopy (AFM), X-ray photoelectron spectroscopy (XPS), and time-of-flight secondary ion mass spectrometry (TOF-SIMS). Experimental details are described in the Supplementary information.

## Results and Discussion

**Figure 3:**
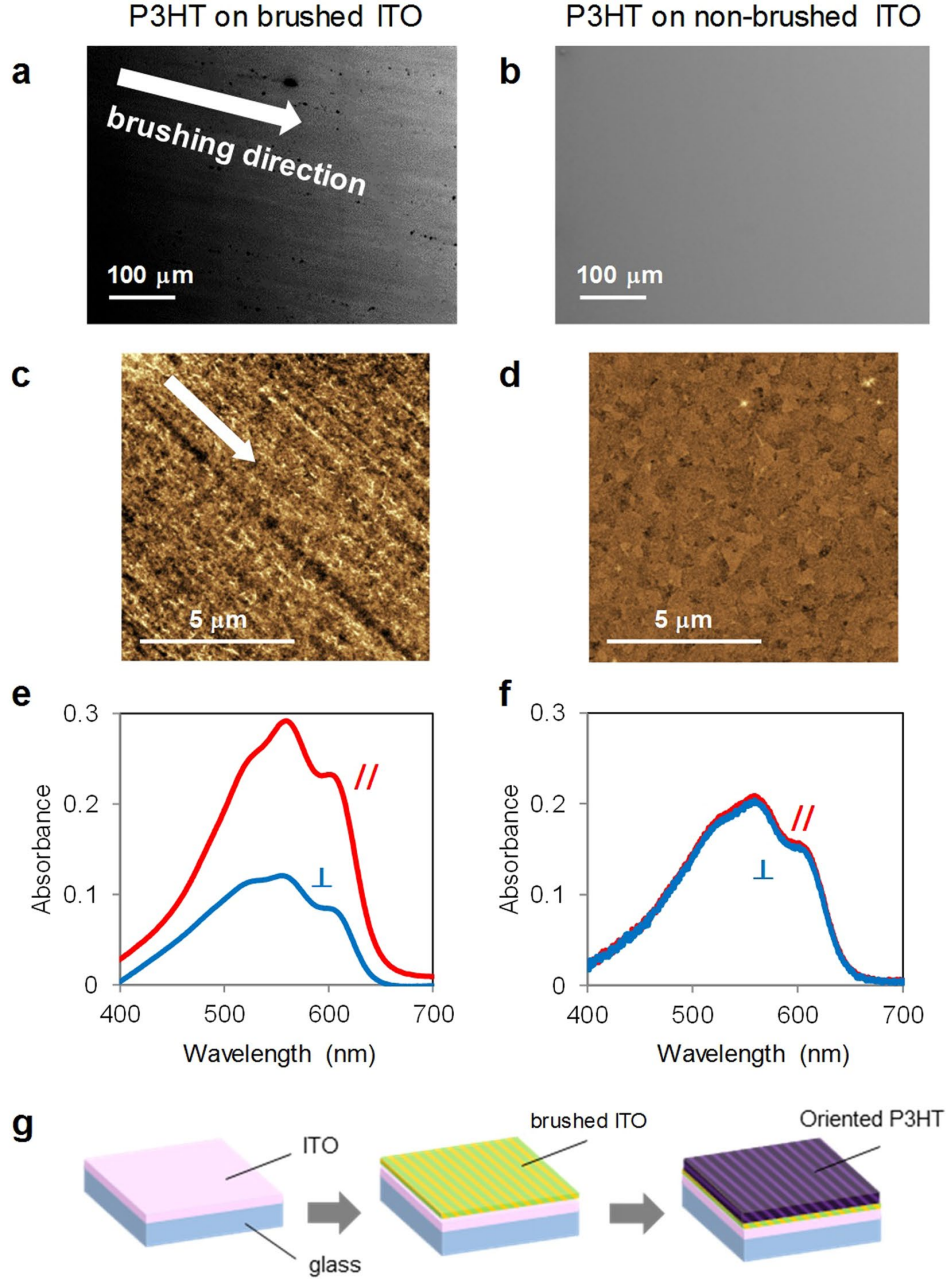
SEM images of P3HT thin films on (**a**) brushed and (**b**) non-brushed substrates. AFM images of P3HT thin films on (**c**) brushed and (**d**) non-brushed substrates. Polarized absorption spectra of P3HT films on (**e**) brushed and (**f**) non-brushed substrates. Red and blue spectra were obtained using incident light polarized parallel (//) and perpendicular (⊥) to the brushing direction, respectively. (**g**) Schematic illustration of sample preparation.

Figure 3a and b show SEM images of P3HT films spin-coated on brushed and non-brushed substrates, respectively. An anisotropic structure in the former film was observed as numerous lines along the brushing direction, whereas a smooth surface without lines was observed in the latter film. The anisotropic feature was also confirmed from AFM observations, as shown in Fig. 3c and d. Figure 3e and f show polarized absorption spectra for P3HT films on brushed and non-brushed substrates, respectively. The parallel (//) or perpendicular (⊥) symbols represent the polarization of light with respect to the brushing direction. The absorbance with the // configuration was greater than that with the ⊥ configuration. This indicates that the π-conjugated backbones of P3HT molecules are aligned along the brushing direction because the direction of the transition dipole moment, HOMO-LUMO transition of P3HT, is parallel to the backbone of the P3HT molecule^37, 38^. The dichroic ratio (*A*_//_/*A*_⊥_) was estimated to be 2.4. The orientation factor, *S* = (*A*_//_ − *A*_⊥_)/(*A*_//_ + *A*_⊥_), was obtained as 0.4. Based on these values, the P3HT backbones are aligned in the in-plane direction. This in-plane alignment was also confirmed by GIXD measurement (Fig. S1 in Supplementary Information). The proposed method is not conducted at high temperature (140–180 °C), such as with conventional frictional transfer, but is established at room temperature. In addition, the molecular orientation is established not by a hard material such as PTFE but using a soft material such as a fabric. Therefore, it is referred to as a soft friction transfer method.

**Figure 4:**
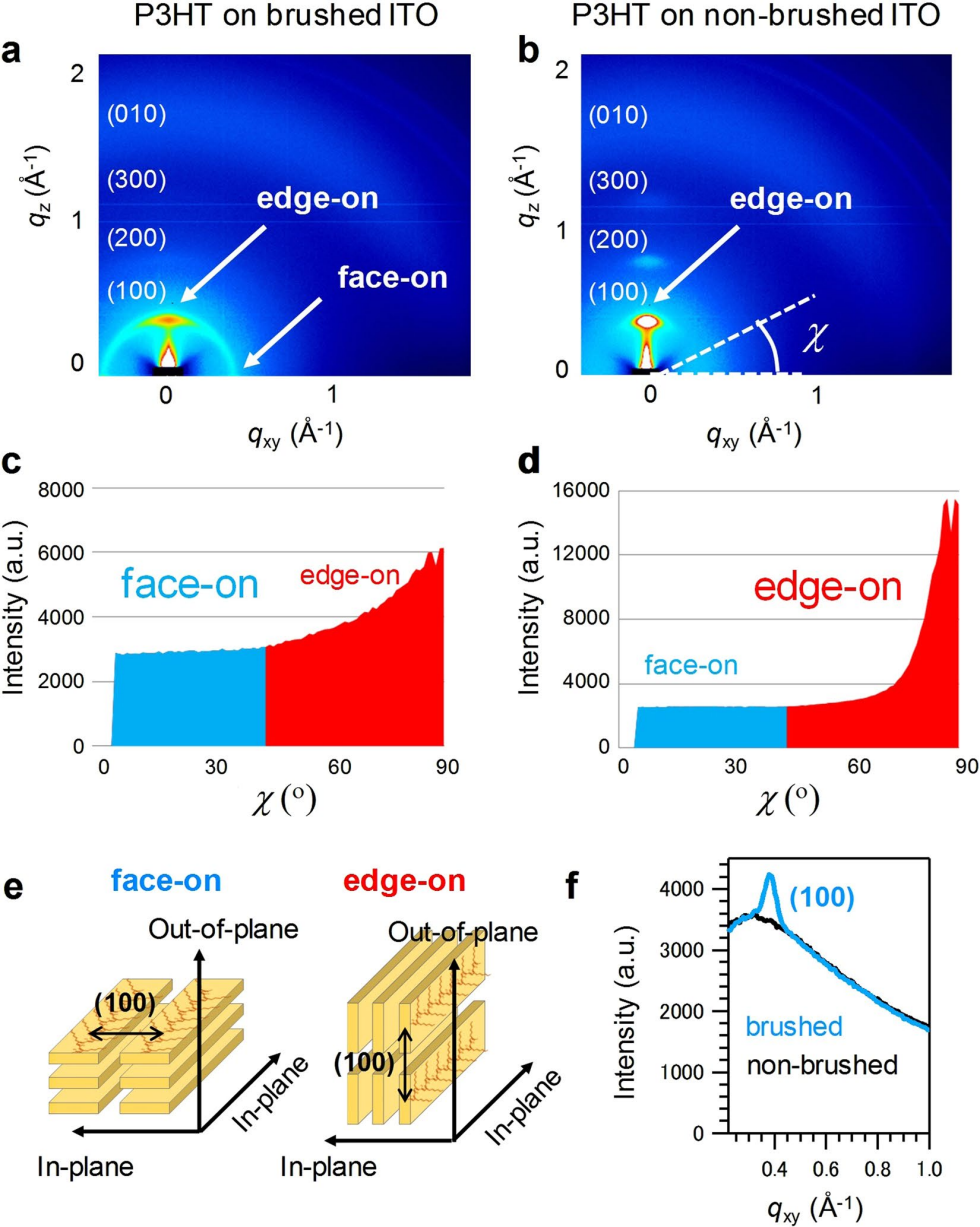
(**a**,**b**) GIXD patterns of P3HT thin films, and (**c**,**d**) intensities of (100) peaks as a function of the pole angle*χ*, for films on (**a**,**c**) brushed and (**b**,**d**) non-brushed substrates. The degrees of face-on and edge-on orientations correspond to the integrated intensities of *χ* = 0 to 45° and *χ* = 45 to 90°, respectively. (**e**) Schematic illustration of face-on and edge-on orientations. (**f**) GIXD intensity profile of the (100) diffraction along *q*_xy_ obtained by integration from *χ* = 0 to 10°.

To investigate the orientation mechanism, the structures of P3HT films on brushed and non-brushed substrates were measured using GIXD, and the results are shown in Fig. 4a and b, respectively. The (100) diffraction of P3HT was observed for both films. The pole angle (*χ*) dependence of the (100) diffraction intensities for the films on brushed and non-brushed substrates is shown in Fig. 4c and d, respectively. The intensities for *χ* = 0 to 45° and *χ* = 45 to 90° denote face-on and edge-on orientations, respectively^39–45^, as shown in Fig. 4e. Therefore, after brushing of the substrate, the edge-on orientation decreases and the component of face-on orientation increases relatively. The diffraction peak for the face-on component emerges in the P3HT film on brushed substrate, as shown in Fig. 4f. Then, major component is also the edge-on orientation in the P3HT film on the brushed substrate, as shown in Fig. 4c.

**Figure 5:**
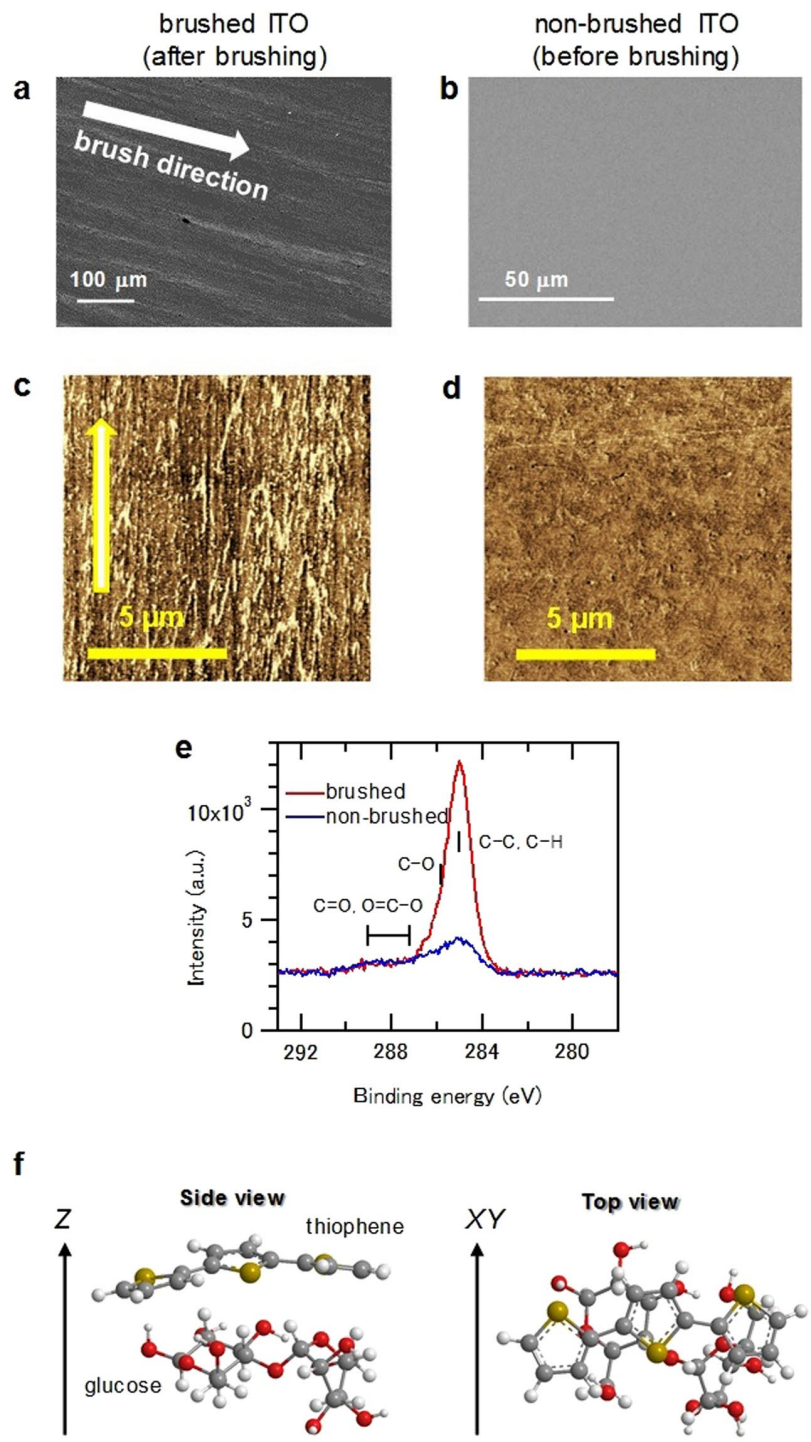
SEM images of (**a**) brushed and (**b**) non-brushed substrates. AFM phase images of (**c**) brushed (**d**) non-brushed substrates. (**e**) XPS spectra of C 1 s on the surfaces of brushed (red) and non-brushed (blue) substrates. (**f**) Relative configuration of optimized structure, obtained using the Gaussian 2009 package^46^ with MP2/6-31 g (d,p) level. Red, grey, white, and yellow spheres represent oxygen, carbon, hydrogen, and sulphur atoms, respectively. *Z* and *XY* represent the out-of-plane and in-plane directions, respectively, when thiophene is present on glucose.

To discuss the orientation mechanism, the substrates were examined before and after brushing using SEM, AFM, XPS, and TOF-SIMS. Figure 5a and b show SEM images of the brushed and non-brushed substrates. There were no features evident before brushing, whereas anisotropic lines along the brushing direction were observed after brushing. The AFM phase images in Fig. 5c and d show anisotropic structures with submicron-meter intervals after brushing. According to the characteristics of the AFM phase image, different colors correspond to different materials on the substrate^24^. XPS measurements of the substrate surfaces indicated an organic compound on the substrate after brushing, as shown in Fig. 5e. This is attributed to cellulose, which is consistent with the TOF-SIMS results (Fig. S2). These results indicate that cellulose is transferred from the velvet fabric to the substrate surface and acts as a template for the alignment of P3HT molecules. Quantum chemical calculations^46^ were also conducted to evaluate the configuration between P3HT and cellulose. The systems used for the calculation were a thiophene trimer and glucose dimer to reduce calculation time, because both P3HT and cellulose are polymers composed of a large number of atoms. As a result, the optimized structure of the system was determined to be the face-on configuration of thiophene aligned on glucose, as shown in Fig. 5f. This structure corresponds to the face-on configuration of P3HT increased by brushing with the velvet fabric on the substrate, as shown in Fig. 4. To conduct further calculations with respect to the film structure, molecular dynamics (MD) simulations for many P3HT molecules would be useful because intermolecular π–π interaction is important to determine the microstructure in P3HT films^47^. However, there have been no reports on calculations of the molecular structures for the orientations of both small and polymer molecules on any brushed substrates, and there have been no reports on such calculations for rubbing studies yet.

**Figure 6:**
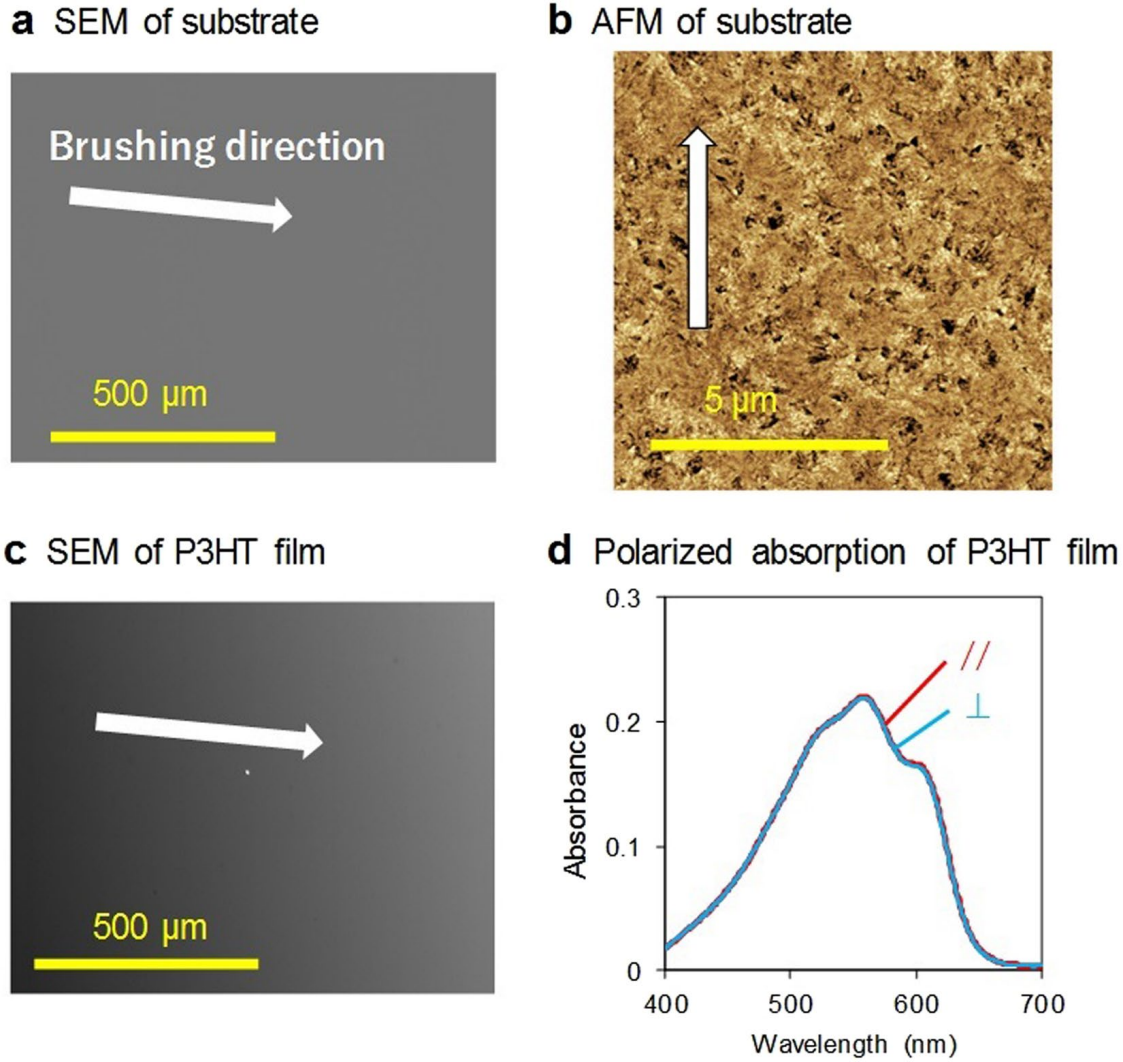
(**a**) SEM and (**b**) AFM images of the substrate washed after brushing. The white arrow indicates the brushing direction. (**c**) SEM image and (**d**) polarized absorption spectra of P3HT film prepared on the substrate washed after brushing. All results showed no anisotropic features.

To confirm the effect of cellulose, several experiments were conducted using a brushed substrate that was then washed with detergent solution. However, neither the anisotropic feature nor the orientation of P3HT was observed (Fig. 6). Therefore, it was concluded that the orientation of P3HT is caused by a chemical process and not a physical process such as mechanical scratching of the glass substrate. Oriented P3HT film was also confirmed to occur with other glass substrates (Fig. S3, Table S1). In addition, the orientations of four other materials prepared by the soft friction transfer method were also observed; poly(2-methoxy-5-(2-ethylhexyloxy)-1,4-phenylene vinylene) (MEH-PPV), poly(9,9-di-n-octylfluorenyl-2,7-diyl) (PFO), poly(9,9-bis-(2-ethylhexyl)-9H-fluorene-2,7-diyl) (PFO2), and Rhodamine 6 G (Fig. S4). The common structure for these molecules and P3HT is a π-conjugated system, which results in attractive interaction between the negative charges of π-electrons and the positive charges of cellulose (Fig. S5).

**Figure 7:**
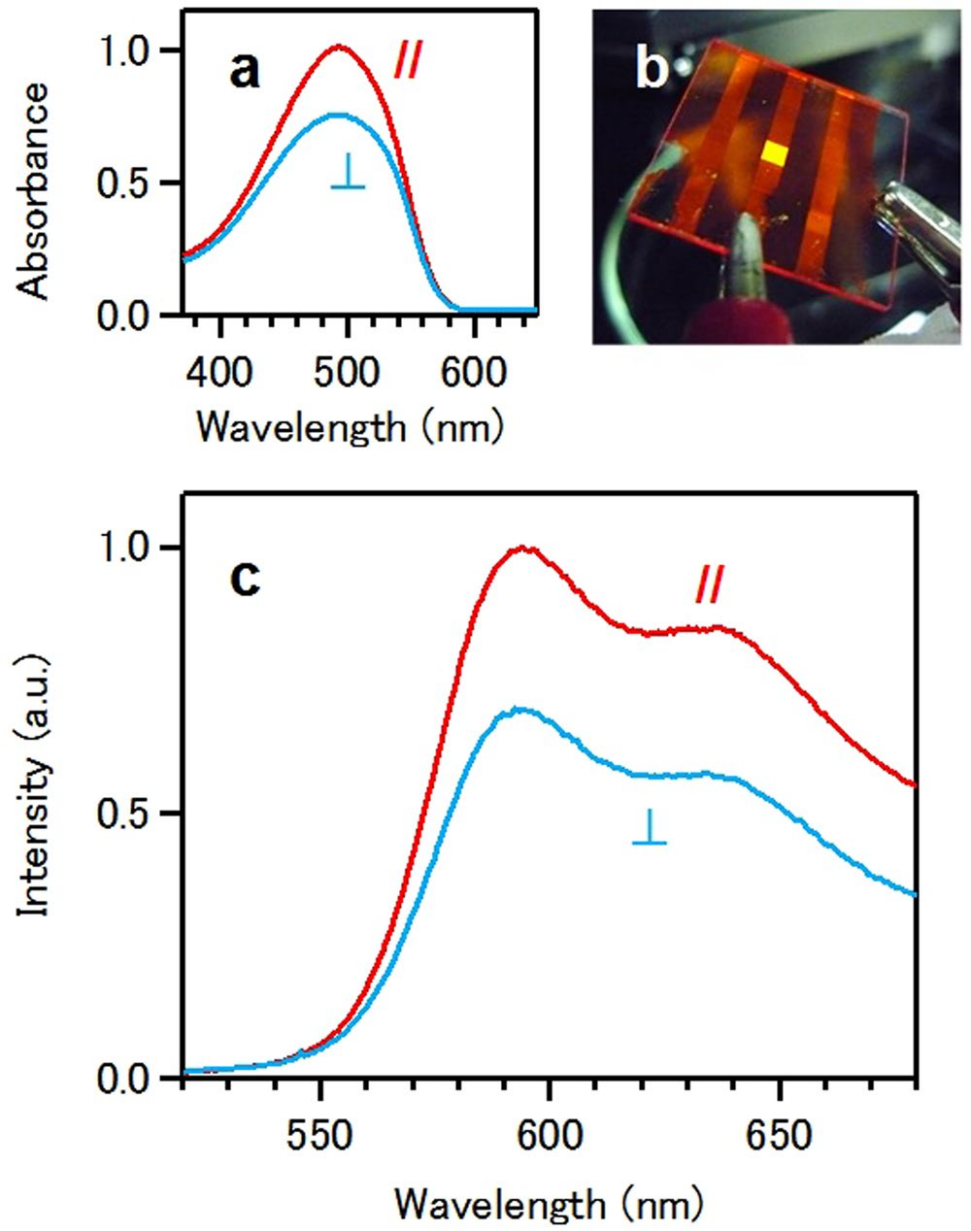
Polarized light-emitting device as an application using the soft friction transfer method. (**a**) Polarized absorption spectra of MEH-PPV film prepared by the soft friction transfer method. Red and blue spectra represent the spectra obtained using incident light polarized parallel (//) and perpendicular (⊥) to the brushing direction, respectively. (**b**) Photograph of the device composed of Al/MEH-PPV/ITO/glass-substrate. (**c**) Polarized EL spectra of the device. Red and blue spectra represent the spectra of light polarized parallel (//) and perpendicular (⊥) to the brushing direction, respectively.

Here, we demonstrate an example of a device application using the soft friction transfer method. Figure 7 shows the polarized electroluminescence (EL) from a light-emitting device. The parallel (//) or perpendicular (⊥) symbols represent the polarization of EL with respect to the brushing direction. The active layer of the device is a MEH-PPV film prepared by the soft friction transfer method. The film with anisotropic structure produces polarized EL. This device was developed by depositing an Al cathode onto the MEH-PPV active layer prepared by the soft friction transfer method.

**Figure 8:**
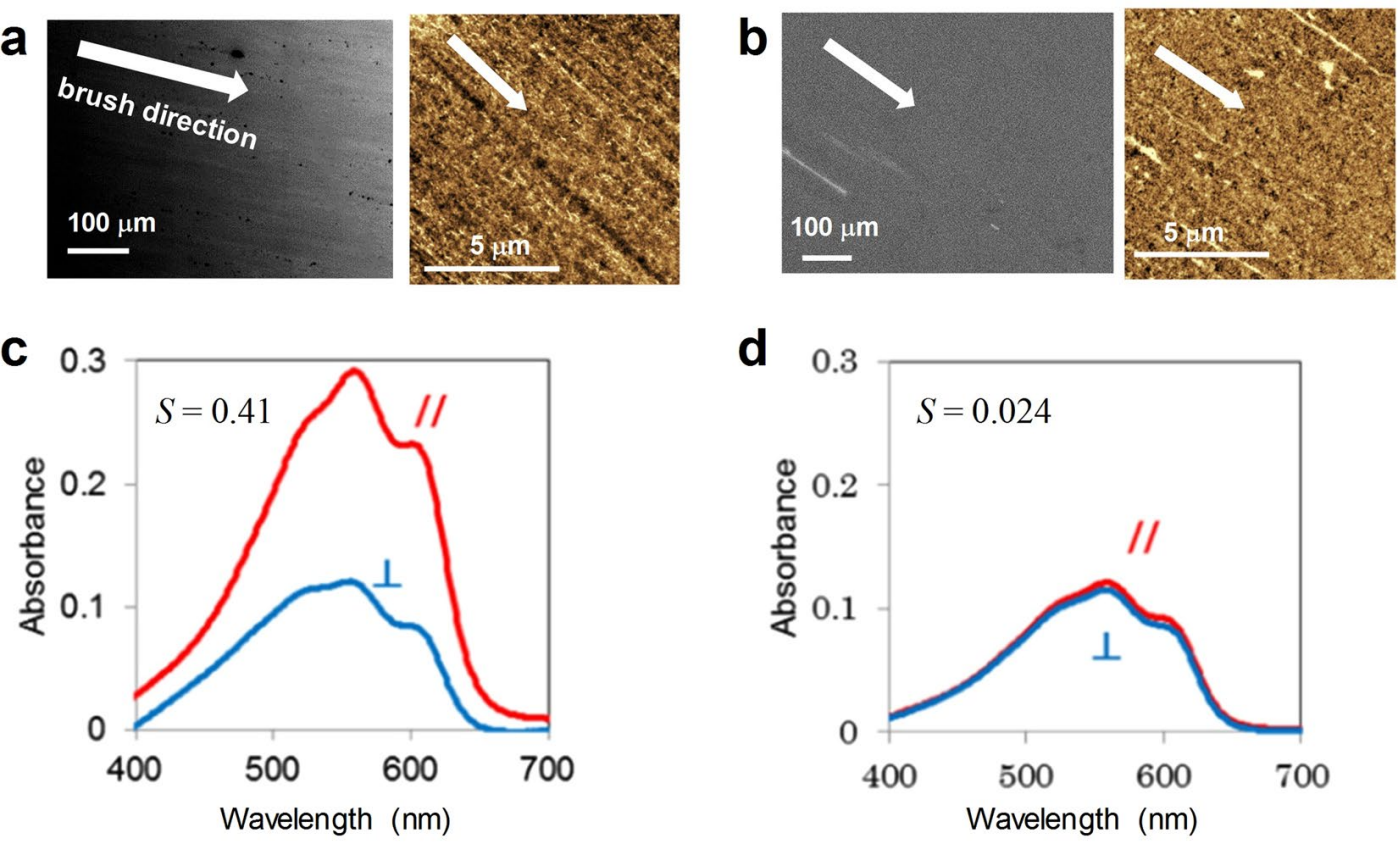
(**a**,**b**) SEM and AFM images, and (**c**,**d**) polarized absorption spectra. (**a**,**c**) P3HT film prepared on ITO brushed by velvet fabric as the soft friction transfer method. (**b**,**d**) P3HT film prepared on ITO brushed by PTFE plate as the conventional friction transfer method; ITO substrate was brushed with PTFE plate heated at 120 °C and P3HT solution was then spin-coated onto the substrate at room temperature.

Finally, the film prepared by the soft friction transfer method was compared with that prepared using a conventional friction transfer method. Briefly, a substrate was brushed with a polytetrafluoroethylene (PTFE) plate heated at 120 °C and P3HT solution was spin-coated onto the substrate at room temperature. This condition is the same as that for soft friction transfer, except for the brushing material and temperature. In addition, anisotropic structures on the substrate after brushing with the PTFE plate were observed using AFM and SEM, as shown in Fig. 8a and b. Figure 8c and d show polarized absorption spectra for the P3HT films prepared by soft friction transfer and by conventional friction transfer with a PTFE plate, respectively. A larger absorbance difference between the // and ⊥ configurations was achieved by the soft friction transfer method. Thus, the soft friction transfer method used in the present study produces P3HT film with higher orientation than that by the conventional friction transfer method; a comparison between the soft friction transfer and the previous study on the conventional friction transfer is given in the Supplementary Information. The soft friction transfer method is thus confirmed to be a powerful method to obtain oriented films at room temperature by a facile process.

## Conclusions

The uniaxial alignment of P3HT thin film was achieved by the soft friction transfer method. The in-plane molecular orientation and out-of-plane face-on orientation were quantified by analysis of absorption spectra and GIXD data, respectively. The orientation mechanism for the P3HT film was analyzed using various methods and was attributed to a chemical process, i.e., cellulose molecules attach to the substrate and act as a template for molecular alignment. The soft friction transfer method does not require high temperature, a glove box, or vacuum equipment, but provides uniaxial oriented films by a facile process that involves brushing a soft velvet fabric on the substrate with a force such as 1–2 kgf/cm^2^ at room temperature. In addition, an oriented film can be picked up by dissolving cellulose on the substrate with water and transferring to another substrate. A film prepared by soft friction transfer was used in a light-emitting device as a representative application, and polarized EL was observed as a result. The soft friction transfer method is thus a versatile method that can be applied to next-generation flexible electronic devices such as transistors, light-emitting diodes, circuits, and sensors.

## Electronic supplementary material

Supplementary Information
