# Supplementary material for: Uniaxial orientation of P3HT film prepared by soft friction transfer method
Source: Sci Rep. 2017 Jul 11;7:5141. doi: 10.1038/s41598-017-05396-9 (PMC5505972; doi:10.1038/s41598-017-05396-9)
Supplement: Supplementary file 1 — Supplementary Information [file 41598_2017_5396_MOESM1_ESM.doc]

Supplementary information

Uniaxial orientation of P3HT film prepared by

soft friction transfer method

Masayoshi Imanishi,^a^ Daisuke Kajiya,^b^ Tomoyuki Koganezawa,^c^ and Ken-ichi Saitow*^,a,b^

^a^Department of Chemistry, Graduate School of Science, Hiroshima University, 1-3-1 Kagamiyama, Higashi-hiroshima, Hiroshima 739-8526, Japan

^b^Natural Science Center for Basic R&D (N-BARD), Hiroshima University, 1-3-1 Kagamiyama,

Higashi-hiroshima, Hiroshima, 739-8526, Japan

^c^Japan Synchrotron Radiation Research Institute (JASRI), SPring-8, 1-1-1 Kouto, Sayo, Hyogo 679-5198, Japan

* Electronic mail: saitow@hiroshima-u.ac.jp

Experimental details:

P3HT thin films and ITO glass substrates were investigated using SEM, polarized absorption spectra, AFM, GIXD, XPS, and TOF-SIMS measurements. SEM images were observed using a scanning electron microscope (S-3400N, Hitachi) with an accelerating voltage of 7–10 kV. Polarized absorption spectra were measured using a UV–Vis spectrophotometer (V-660, Jasco) and a polarizer. To obtain the absorption spectrum, transmission spectra were measured for the sample (P3HT film on a substrate), reference (substrate), and dark signals. The absorption spectrum of the P3HT film was obtained using these three signals, based on the Beer-Lambert law. The polarizer was placed in front of the sample. The orientation factor, *S*, was obtained from absorbance at parallel and perpendicular configuration. *S* = 1 and 0 represent complete uniaxial alignment and a random isotropic distribution, respectively. AFM images was measured with an atomic force microscope (SPM-9700, Shimadzu) in tapping mode using a microcantilever (OMCL-AC200TS, Olympus) with a spring constant of 9 N/m and a resonance frequency of 150 kHz. GIXD measurements were conducted at the BL19B2 beamline of SPring-8 with an X-ray energy of 12.39 keV (*λ* = 1 Å). X-rays were irradiated at an incident angle of 0.12° and the scattered X-rays were recorded using a 2-D image detector (Pilatus 300K, Dectris). The direction of incident X-rays was parallel to the brushing direction. XPS was performed using a spectrometer (Quantera SXM, ULVAC-PHI) with monochromatized Al K_α_ (1486.6 eV) as the X-ray source and with a pass energy of 55 eV. TOF-SIMS was performed using a spectrometer (TOF.SIMS300, ION-TOF) with Bi^+^ ions at an accelerating voltage of 25 kV irradiated onto the sample surface and the secondary ions detected by the time-of-flight method.

The transfer of oriented film to another substrate was performed as the following procedures. The film on the substrate was immersed in water with small amount of surfactant, whose temperature was set to be 60 ^o^C. After several tens of minutes, the P3HT film was peeled from the substrate and floated in water. Then, the P3HT film was putted on a glass slide.

The EL spectra of a device composed of Al/MEH-PPV/ITO/glass-substrate were measured using a commercial spectrometer instrument (T64000, Horiba Jobin Yvon). The MEH-PPV layer was prepared by soft friction transfer method. Namely, ITO-coated glass substrate was brushed using a velvet fabric at room temperature and MEH-PPV solution was then spin-coated on the substrate. The Al cathode was deposited on MEH-PPV layer using a vacuum evaporation system (SVC-700TM, Sanyu Electron). EL from the device was observed by applying voltage using alkaline batteries. Polarized EL spectra were measured by placing a polarizer in front of the device and by rotating the device. All the EL spectra were calibrated using an instrument function, which was obtained from an NIST standard halogen lamp (LS-1-CAL, Ocean Optics).

Detail of comparison between conventional and soft friction transfer methods:

The conventional friction transfer at room temperature using a PTFE plate did not give an orientation of molecules, while the soft friction transfer at room temperature using fabric gave an orientation of molecules. An essential factor of the soft friction transfer is a molecular transferred from a soft material to the substrate by a weak force at room temperature. The fabric of soft material transfers cellulose on the substrate and acts as a template for molecular orientation, while PTFE plate requires high temperature to transfer PTFE on the substrate.

The previous study^1^ shows the orientation by sliding a PTFE plate on a substrate, i.e., oriented films with the absorption dichroic ratio of 3.0 for *p*-sexithiophene and 4.1 for PPV, and so on. The reason for no orientation of P3HT using a PTFE plate in the present study is due to experimental conditions. Namely, the previous study slid the PTFE plate on the substrate at 200 ^o^C and used a drop casting method, whereas the present study slid the PTFE plate on the substrate at 120 ^o^C and used a spin coating method. We slid the PTFE plate on a substrate at 200 ^o^C. However, the P3HT film cannot be prepared by spin coating, owing to no wettability of the P3HT solution on the substrate after sliding the PTFE plate.

Figure S1 shows GIXD results of P3HT film prepared using soft friction transfer method. The parallel (//) or perpendicular (⊥) symbols represent the direction of incident X-ray with respect to the brushing direction. The (100) diffraction peak of P3HT along *q*_xy_ is observed at // configuration and is not observed at ⊥ configuration. This result indicates that P3HT backbones are aligned along the brushing direction.


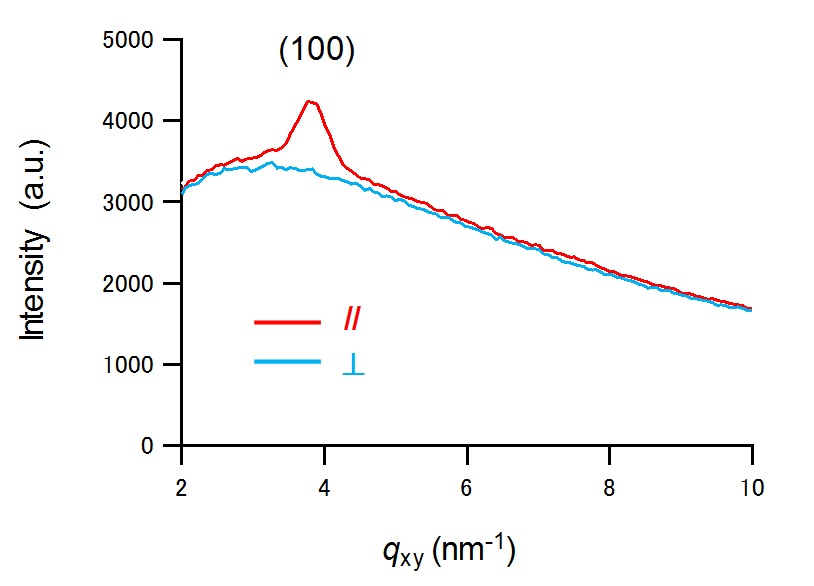


**Figure S1**. GIXD intensity profile of the (100) diffraction of P3HT along *q*_xy_, obtained from the integration from *χ* = 0 to 10^o^. The P3HT film was prepared using soft friction transfer method. Red and blue spectra were obtained with parallel (//) and perpendicular (⊥) configurations, respectively, i.e., the direction of incident X-ray was // or ⊥ to the brushing direction.

Figure S2a shows TOF-SIMS results for the brushed substrate. Fragment ions were observed at around *m*/*z* 59, 71, 87, and 99. The fragment ions were similar to those of cellulose, as shown in Fig. S2b, and were distributed along the brushing direction, as shown in Fig. S2c. These results indicate that the cellulose is attached onto the substrate by brushing. Cellulose was also detected from the velvet fabric, as shown in Fig. S2d, which implies that the cellulose was transferred from the velvet fabric to the substrate.


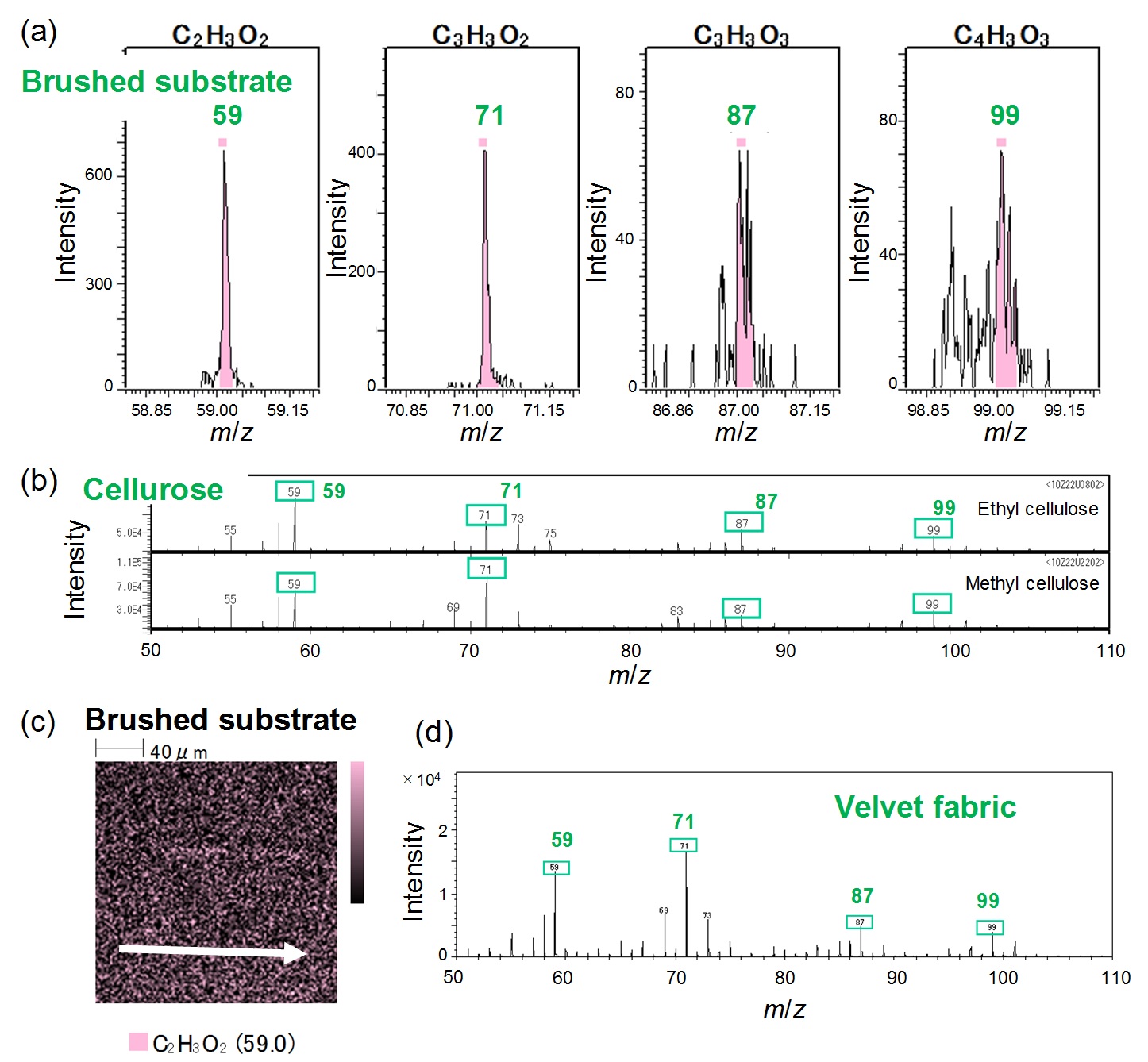


**Figure S2**. TOF-SIMS results for (a) the brushed substrate and (b) cellulose. (c) TOF-SIMS mapping result of *m*/z 59.0 for the brushed substrate. Brighter colors indicate a greater amount of *m*/*z* 59.0. The white arrow indicates the brushing direction. (d) TOF-SIMS results for velvet fabric.


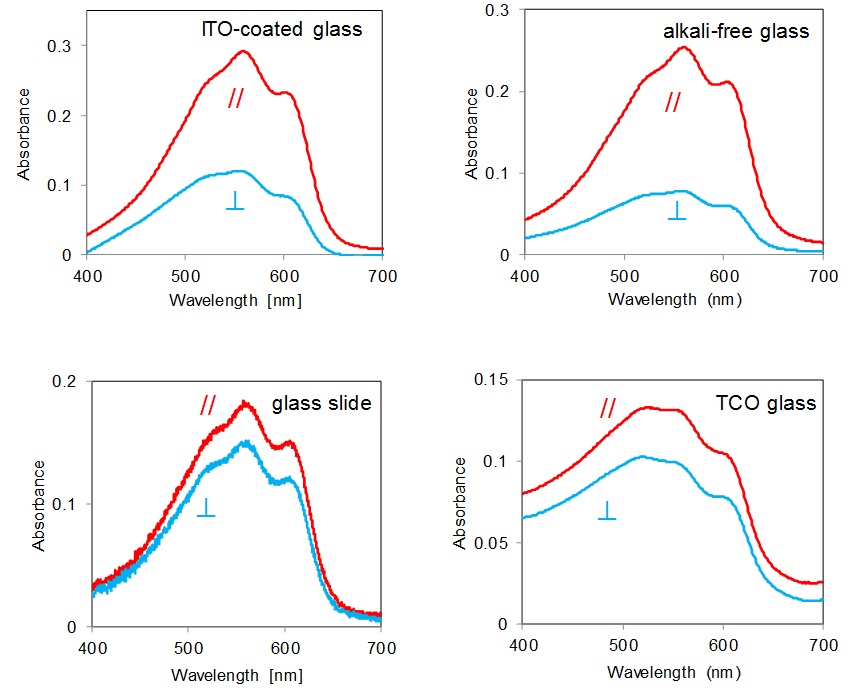


**Figure S3**. Polarized absorption spectra for P3HT films prepared by soft friction transfer on various substrates, i.e., ITO-coated glass (FLAT-ITO, Geomatec), alkali-free glass (NA32SG, AvanStrate), glass slide (Matsunami Glass Ind.), and transparent conductive oxide (TCO) glass (fluorine doped tin oxide (FTO) coated glass, AGC Fabritech). Red and blue spectra were obtained with // and ⊥ configurations, respectively.

**Table S1.** Summary of results, where yes and no represent the confirmation of anisotropic and isotropic structures, respectively.

|  | ITO-coated  glass substrate | glass substrate |
| --- | --- | --- |
| **Un-brushed substrate** |  |  |
| Polarized absorption | no | no |
| SEM image | no | no |
| AFM image | no | no |
| **Brushed substrate** |  |  |
| Polarized absorption | yes | yes |
| SEM image | yes | yes |
| AFM image | yes | yes |
| TOF-SIMS mapping | yes | yes |
| **Brushed and washed substrate** | |  |
| Polarized absorption | no | no |
| SEM image | no | no |
| AFM image | no | no |


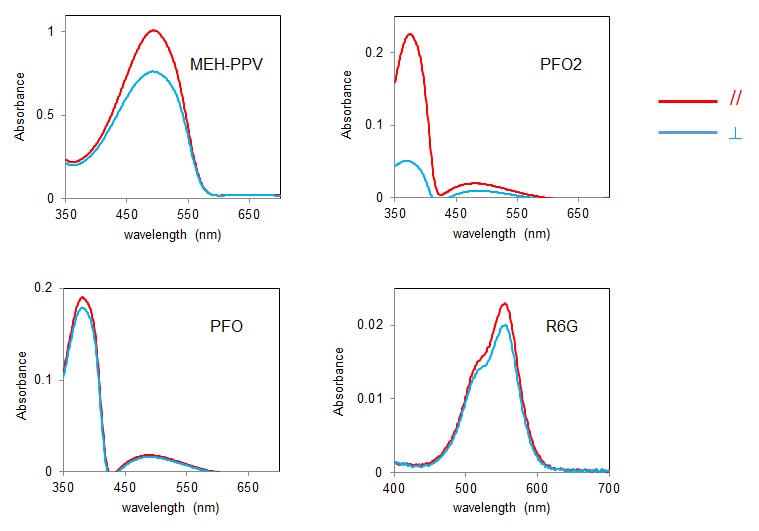


**Figure S4**. Polarized absorption spectra for MEH-PPV, PFO, PFO2, and R6G films prepared by the soft friction transfer method. Red and blue spectra were obtained with // and ⊥ configurations, respectively. The materials used are poly[2-methoxy-5-(2-ethylhexyloxy)-1,4-phenylenevinylene] (MEH-PPV) (541443, Sigma-Aldrich), poly(9,9-di-*n*-octylfluorenyl-2,7-diyl) (PFO) (571652, Sigma-Aldrich), poly[9,9-bis-(2-ethylhexyl)-9H-fluorene-2,7-diyl] (PFO2) (571032, Sigma-Aldrich), and Rhodamine 6G (R6G) (R4127, Sigma-Aldrich).


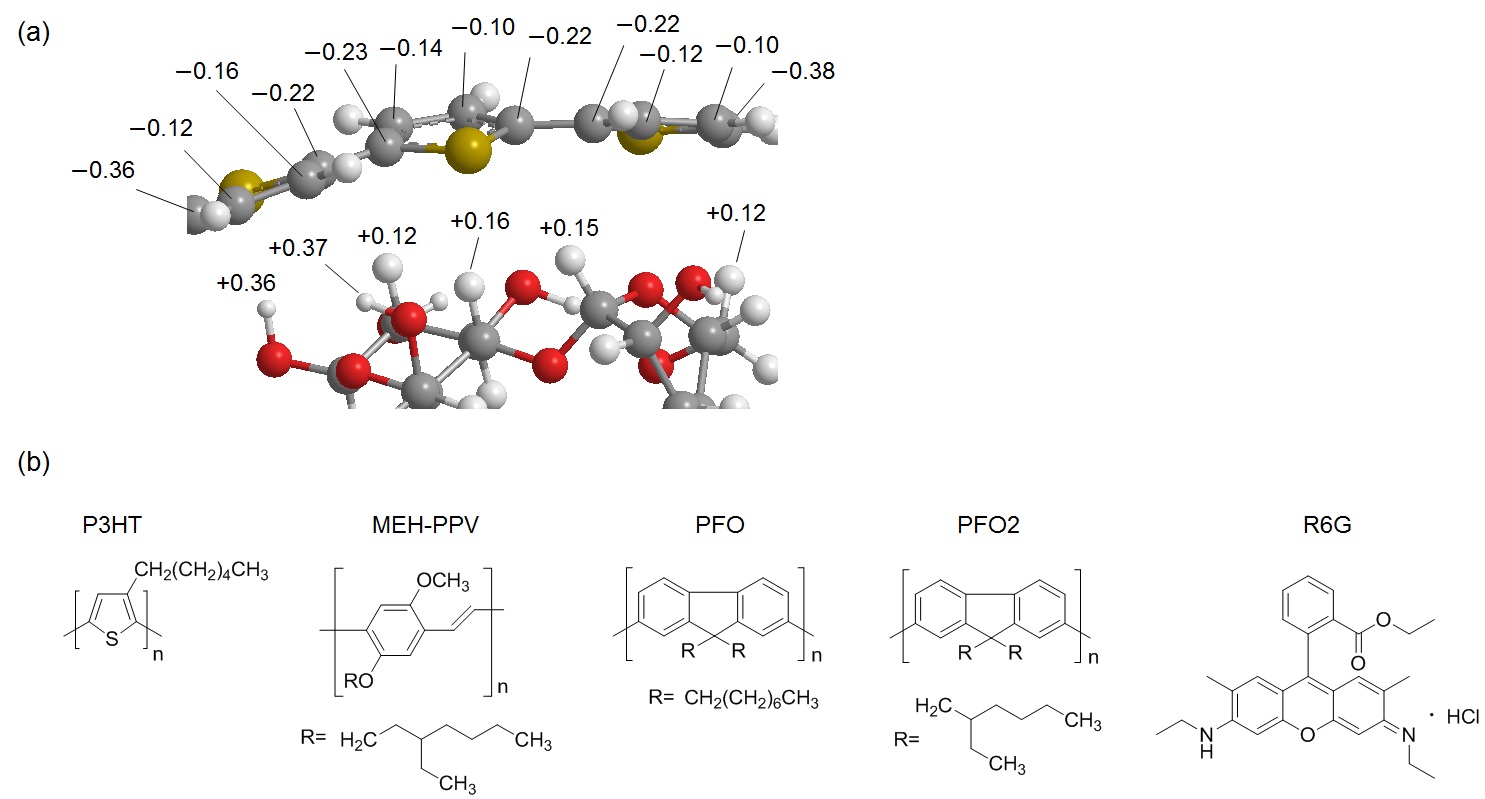


**Figure S5.** (a) Calculated Mulliken charges for OH group and H atom of cellulose and the π-conjugated system of thiophenes at the configuration of Fig. 4f. Calculation was performed by using the Gaussian 2009 package^2^ with MP2/6-31g(d,p) level. White and grey spheres represent hydrogen and carbon atoms, respectively. (b) Chemical structures of materials used in the present study.

**REFERENCES**

1. Chen, X. L., Bao, Z., Sapjeta, B. J., Lovinger, A. J. & B. Crone B. Polarized Electroluminescence from Aligned Chromophores by the Friction Transfer Method. *Adv. Mater*. **12**, 344−347 (2000).
2. Frisch, M. J., Trucks, G. W., Schlegel, H. B. et al., GAUSSIAN 09, Revision B.01, Gaussian, Inc., Wallingford, CT, (2010).
